# TIPMaP: a web server to establish transcript isoform profiles from reliable microarray probes

**DOI:** 10.1186/1471-2164-14-922

**Published:** 2013-12-27

**Authors:** Neelima Chitturi, Govindkumar Balagannavar, Darshan S Chandrashekar, Sadashivam Abinaya, Vasan S Srini, Kshitish K Acharya

**Affiliations:** 1 Institute of Bioinformatics and Applied Biotechnology (IBAB), Biotech Park, Electronic City, Bengaluru (Bangalore) 560 100, Karnataka state, India; 2 Research Scholar, Center for Computational Natural Sciences and Bioinformatics (CCNSB), International Institute of Information Technology (IIIT-H), Gachibowli Hyderabad 500 032, Andhra Pradesh, India; 3 Research Scholar, Manipal University, Manipal 576 104, Karnataka, India; 4 Shodhaka Life Sciences Pvt. Ltd., IBAB, Biotech Park, Electronic City, Bengaluru (Bangalore) 560 100, Karnataka state, India; 5 Ankur Healthcare Pvt Ltd., Rajaji Nagar, Bengaluru (Bangalore) 560 010, Karnataka state, India

**Keywords:** Alternative splicing, Alternatively spliced, Microarray, Affymetrix, Azoospermia, Transcript isoforms, mRNA isoforms, Transcriptome, Gene expression

## Abstract

**Background:**

Standard 3′ Affymetrix gene expression arrays have contributed a significantly higher volume of existing gene expression data than other microarray platforms. These arrays were designed to identify differentially expressed genes, but not their alternatively spliced transcript forms. No resource can currently identify expression pattern of specific mRNA forms using these microarray data, even though it is possible to do this.

**Results:**

We report a web server for expression profiling of alternatively spliced transcripts using microarray data sets from 31 standard 3′ Affymetrix arrays for human, mouse and rat species. The tool has been experimentally validated for mRNAs transcribed or not-detected in a human disease condition (non-obstructive azoospermia, a male infertility condition). About 4000 gene expression datasets were downloaded from a public repository. ‘Good probes’ with complete coverage and identity to latest reference transcript sequences were first identified. Using them, ‘Transcript specific probe-clusters’ were derived for each platform and used to identify expression status of possible transcripts. The web server can lead the user to datasets corresponding to specific tissues, conditions via identifiers of the microarray studies or hybridizations, keywords, official gene symbols or reference transcript identifiers. It can identify, in the tissues and conditions of interest, about 40% of known transcripts as ‘transcribed’, ‘not-detected’ or ‘differentially regulated’. Corresponding additional information for probes, genes, transcripts and proteins can be viewed too. We identified the expression of transcripts in a specific clinical condition and validated a few of these transcripts by experiments (using reverse transcription followed by polymerase chain reaction). The experimental observations indicated higher agreements with the web server results, than contradictions. The tool is accessible at http://resource.ibab.ac.in/TIPMaP.

**Conclusion:**

The newly developed online tool forms a reliable means for identification of alternatively spliced transcript-isoforms that may be differentially expressed in various tissues, cell types or physiological conditions. Thus, by making better use of existing data, TIPMaP avoids the dependence on precious tissue-samples, in experiments with a goal to establish expression profiles of alternative splice forms – at least in some cases.

## Background

Differential gene expression is a pre-requisite for normal and abnormal cellular/physiological events. While scientists have been keen on characterizing proteomes for every tissue or cell-type, it has been easier to explore expression at the mRNA level. DNA microarrays have been extensively used to list differentially transcribed genes across tissues or conditions. The generated expression data is usually deposited in repositories such as Gene Expression Omnibus (GEO) [1] and ArrayExpress [2]. Many designs of microarrays have been in use, among which Affymetrix chips have been popular.

Most of the microarray based gene expression studies focused on identification of differentially regulated ‘genes’ , but not their ‘alternatively spliced transcript isoforms’. About 95% of human genes undergo alternative splicing [3] and there are evidences for association of alternatively spliced forms with diseases/disorders [4-7]. These mRNA-forms can be potential targets for disease treatments as well [5,8,9]. Hence, it is important to catalogue expression of specific transcripts for as many genes as possible.

While exon arrays [10-12] and RNA-sequencing [13,14] are being used for expression profiling of alternatively spliced forms, it is possible to identify the expression status of some transcripts using gene expression data, which already exists for a huge variety of tissues and conditions, across different species. This can be relatively more effective with standard 3′ Affymetrix microarrays due to the following reasons: a) the average number of probes per gene/transcript is higher [15], b) it is possible to get absolute calls (transcribed/not-detected), which are compatible with our recent algorithm for deriving a consensus expression status across multiple hybridizations/studies [16] and c) these microarrays are particularly the most commonly used platforms (specific statistics in results and discussion sections). In fact, scientists have used such data and identified differentially regulated alternatively spliced forms. They realigned the probe sequences to transcripts from National Center for Biotechnology Information (NCBI), Ensembl, UniGene or AceView and analyzed the data. But such efforts have been limited to a few tissues or conditions [17-21]. Brainarray database has realigned probes to different transcripts, and generated files with probe-clusters that include transcript-specific probes as well as those common to multiple alternatively spliced forms [22]. Since these two types of probes cannot be easily identified, expression profiles obtained using these files might not be very useful for identifying transcript-specific expression status. A few algorithms were also developed to tap the transcript level expression data from gene arrays [12,23,24]. All these previous efforts show that the already existing expression data from standard Affymetrix gene arrays can be used to obtain insights into differential regulation of specific transcript isoforms in different conditions and tissues. However, there is no online tool available to facilitate such analysis. In addition, it is becoming evident that not all the previously used microarray probes are of good quality. Earlier reports have shown that, many microarray probes do not have 100 percent identity, along their entire length, to any transcript [25]. There are also evidences of improved accuracy in microarray results on removal of probes with improper annotations [26]. We developed TIPMaP to fill the current void. Further, we experimentally validated the results obtained from this tool for a few transcripts relevant to the non-obstructive azoospermia (NOA) disease.

## Implementation

### Probe and transcript sequences

RefSeq transcript sequences (FASTA format) for human [27], mouse [28] and rat [29] were downloaded from NCBI. Probe sequences for 3′ gene expression arrays (FASTA format) were downloaded from Affymetrix [30].

### Gene expression data

Raw gene expression data files (.CEL), produced using 31 standard 3′ human, mouse and rat Affymetrix platforms are being downloaded from GEO [1]. About 4000 experiments have been downloaded on priority. In addition, a provision has been created for automatic downloading as per user’s need.

### Perform BLAST

Basic Local Alignment Search Tool (BLAST) version 2.2.25 was downloaded from NCBI [31]. Local RefSeq transcript databases, for human, mouse and rat species, were set up for BLAST operation, using formatdb program. The blastall program was used to align each platform’s probe sequences to local transcript databases.

### Generate transcript specific probe-clusters

Alignment results were parsed to identify probes that map to each transcript. In a significant number of cases one or more probes showed a reliable match to only one splice variant; these probes uniquely mapped to specific transcripts with 100 percent sequence identity, and along their complete length (25 bases). Using such probes, ‘transcript-specific probe-clusters’ were derived and stored as a new chip definition file (CDF), for every platform considered. Such ‘transcript-specific probe-clusters’ were derived for single-transcript-genes and multi-transcript-genes.

### Process gene expression data

Bioconductor’s makecdf package [32] was used to make CDF environments for every new CDF file. These new CDF environments, Microarray Suite 5.0 (MAS5) and Robust Multi-Array Average (RMA) algorithms of Bioconductor’s affy [33] and simpleaffy packages [34] were used for data normalization. RMA-normalized gene expression data is used to list differentially regulated transcripts across the conditions or groups, chosen by the user. For every transcript in a group, an average signal intensity is calculated (across hybridizations or GSMs in a group selected by the user) and a t-test is performed, using CPAN modules such as Statistics::TTEST [35] and Statistics::Distributions [36] to identify transcripts, whose average signal intensities are significantly different (p-value < = 0.05) between the groups or GSM-sets identified by the user. MAS5 processed gene expression data is used to derive differentially detected transcripts, using probe-cluster detection calls. A probe-cluster is labeled as ‘present’ (if the p-value derived is equal to or less than 0.05), ‘absent’ (p-value is equal to or greater than 0.065) and ‘marginal’ (p-value is greater than 0.05 and less than 0.065). For every transcript in a group, a consensus call is derived across GSMs selected by the user. A transcript is labeled as ‘transcribed’ , if majority of GSMs or hybridizations have ‘present’ as detection call. For example, a transcript reported to be transcribed in 4 hybridizations and not-detected in one hybridization, would be shown as transcribed with a percentage detection of 80. Similarly, a transcript is labeled as ‘not-detected’ , if majority of GSMs or hybridizations have ‘absent’ as detection call and a transcript is labeled as ‘no-call’ , if there are equal present and absent calls.

### Scripts and programs

Perl scripts were written to identify and group the transcript-specific probes, create new CDF files and predict differentially regulated or detected transcripts. R scripts were written and R packages were used to process raw gene expression data. Perl, HTML and CGI scripts were used in interface development.

### Experimental validation by reverse transcription PCR

#### Transcript selection

Data from a specific study (E-TABM-234, ArrayExpress [2]) on gene expression profiling, which used Affymetrix chip (human genome U133 plus 2.0 array), was processed using TIPMaP (‘process data’ option in the interface). Expression at transcript level was obtained using the data from this study, across the 31 hybridizations for non-obstructive azoospermia (Johnsen score counts ranging from 2 to 8). The resulting transcripts were grouped into 10 sets based on the reliability of expression status. The reliability, which reflected the consistency, was in turn determined by the percentage of hybridizations with the same expression status. For example, transcripts in top-set (rank 1) had same expression pattern across 90 or higher % of hybridizations. Similarly, the genes designated as transcribed in non-obstructive azoospermia were also downloaded from MGEx-Tdb [16] and grouped into 10 different reliability ranks. The following selection criteria were then applied:

a) Genes should code for multiple transcripts.

b) Genes should be transcribed as per MGEx-Tdb and should belong to reliability ranks 1–5.

c) Transcripts encoded by these genes should show a differential expression status in the non-obstructive azoospemic condition, as per TIPMaP – i.e., one transcript should be transcribed and the other ‘not-detected’. In addition, the transcripts should belong to reliability ranks 1 to 3 for both transcribed and not-detected status of expression.

d) At least 6 probe-pairs should be used to derive an expression call for a transcript.

#### Experiments

Research on humans was carried out in compliance with the Helsinki declaration and the procedures have been approved by Institutional Biosafety Committee (IBSC), Institute of Bioinformatics & Applied Biotechnology (IBAB) and Ankur hospitals. Testicular biopsy samples were obtained for four non-obstructive azoospermic donors of 30 to 38 years age, who provided consent after being informed about the use of the sample for studies, and were stored in RNA later solution (Ambion, cat no: AM7020). RNA was isolated using RiboPure kit (Ambion, cat no: AM1924). The RNA was confirmed to be of good quality by electrophoretic and spectrophotometric analysis. About 2.2 μg of RNA was used for cDNA synthesis (Thermo scientific, verso cDNA synthesis kit cat no: AB-1453/B). PCR was performed using DyNAzyme II DNA Polymerase (Thermo scientific, cat no: F-501 L) and transcript-specific primers. RT-PCR products were checked on 10% polyacrylamide gel and the gel images were captured using G:BOX Chemi XT 4 instrument (Syngene) [see Additional file 1 for primer details].

### Resource use

Three simple steps have to be followed to identify differentially regulated or detected alternatively spliced forms.

#### Step 1, Choose datasets

Specific GEO series (GSEs) have to be selected to start the analysis. The user can, however, begin the query with one or more GEO series (GSE IDs) or sample identifiers (GSM IDs). If specific GEO datasets have not been identified yet, the user can also begin with genes (official gene symbols) or RefSeq transcript IDs or general keywords. Search by keywords results in display of corresponding GSE IDs and summary of the experiments. When queried with gene or transcripts, the tool displays the GSEs that have been already downloaded, but only those with information about the expression status of the queried gene/transcript. This page also includes expression status (detection status: transcribed, ‘T’; not-detected, ‘ND’; and marginal, ‘M’ and RMA-processed signal intensity) of the queried genes and their transcript IDs. Search by GSM identifier displays GSE ID, GEO platform identifier (GPL ID), title, source name, characteristics and description.

Clicking the displayed GSE ID or search by GSE identifier results in a list of corresponding GSMs along with GPL ID, title, source name, description, characteristics and a check box. The user has to then select the specific GSMs and group them, as shown in step 2 below.

#### Step 2, Create group (s)

To start an analysis, user has to create one (to know absolute expression status) or two (to find the differential expression status) group(s). Samples or GSMs with similar biological conditions can be grouped. A group can be created by selecting the check boxes provided for each GSM, and then by clicking on ‘Group all selected GSMs’. A group can have any number of GSMs and any number of groups can be made, but the tool performs analysis across 2 groups at a time. For example, if three groups are made, the tool compares, and provides results for, groups 1 vs. 2, groups 1 vs. 3 and groups 2 vs. 3.

#### Step 3, Choose expression type

The tool can be queried for differential status (up and/or down regulation) and/or absolute calls (transcribed or not-detected expression status). Since the number of probes that uniquely map to each transcript varies (see Table 1), and since the reliability of the transcription profiles (output) depends on the number of such good probes, an option is provided in the query page to set thresholds for the number of probes.

**Table 1:** Distribution of transcript-specific probes and transcript-specific probe-clusters across platforms

**No. of transcript-specific probes**	**No. of transcript specific probe-clusters**									
	**HG U133 Plus 2**		**Mouse 430 2**		**HG U133 A**		**MG U74 Av2**		**Rat 230 2**	
	STGs*	MTGs**	**STGs**	**MTGs**	**STGs**	**MTGs**	**STGs**	**MTGs**	**STGs**	**MTGs**
1	129	363	120	143	86	185	67	32	150	83
2	104	192	108	69	61	107	70	27	128	42
3	77	106	80	51	40	53	91	22	107	21
4	95	88	103	48	58	39	49	15	129	15
5	83	60	78	28	51	26	42	10	139	13
6	108	68	112	16	82	47	44	9	158	10
7	147	49	131	23	110	22	56	7	184	6
8	252	63	189	32	181	33	55	11	267	19
9	444	87	308	33	365	51	73	7	510	14
10	910	137	617	48	718	77	54	9	1029	33
11	4850	662	7006	398	3475	342	88	12	5973	135
> = 12	4734	383	5007	219	1941	130	5587	219	2565	40
Total no. of transcripts***	***11933***	***2258***	***13859***	***1108***	***7168***	***1112***	***6276***	***380***	***11339***	***431***
***Total no. of genes***	***11933***	***1773***	***13859***	***892***	***7168***	***953***	***6276***	***340***	***11339***	***385***
***Total no. of transcript specific probes***	***195511***	***21305***	***218403***	***11436***	***101603***	***9327***	***102472***	***4473***	***99287***	***1903***

*STGs: transcripts from single-transcript-genes (transcripts to which the probe-clusters uniquely map).

**MTGs: transcripts from multi-transcript-genes (transcripts to which the probe-clusters uniquely map).

***Total number of transcript-specific probe-clusters is same as total number of transcripts.

#### Output

Transcripts with differential ‘regulation’ are listed along with their gene names, RefSeq transcript ID, mean signal intensity for each group, regulation status, fold change and p-value. Transcripts with differential ‘detection’ (absolute calls) are listed along with their gene names, RefSeq transcript ID, detection status and percentage detection. The analysis results can be exported in a tab separated text file. Each RefSeq transcript ID is linked to basic transcript and the corresponding gene and protein. All these information have been downloaded from NCBI and UniProt [37] databases and stored in a MySQL Relational Database Management System (RDBMS). Gene information section displays NCBI ID, official symbol, description, aliases, Entrez-summary, chromosomal location, orientation, size, sequence and OMIM IDs. Protein information provides details for protein isoforms, with corresponding sequence, function, molecular weight and amino acid length. Transcript information includes transcript ID, graphical display of the transcript-structure (exons and introns) and sequences of the corresponding gene, exons and introns. Selected exons, introns or exon-intron junctional sequences can be scanned for experimentally proven branch sites and splice factor binding sites. These experimentally identified sites were taken from SpliceAid2 [38]. In addition, links to RNAanalyzer [39], RESCUE-ESE [40], ESRsearch [41], ACESCAN2 [42] and WebLogo [43] are provided to perform structural and sequence analysis on selected sequences. There are also links to other alternative splicing resources, ProSplicer [44], AceView [45], fast DB [46], ASG [47], SpliceMiner [48] and ASTRA [49]. BLAST result section displays probe to transcript alignment results. Transcript-specific probe-cluster gives details of the probes, which are used to create transcript specific probe-set. In addition, probe’s X and Y co-ordinates, matched start and end positions on the transcript, percentage identity and coverage are also displayed. Expression profile for all transcripts in a GSE (across GSMs) can be viewed using the browse option.

**Figure 1:**
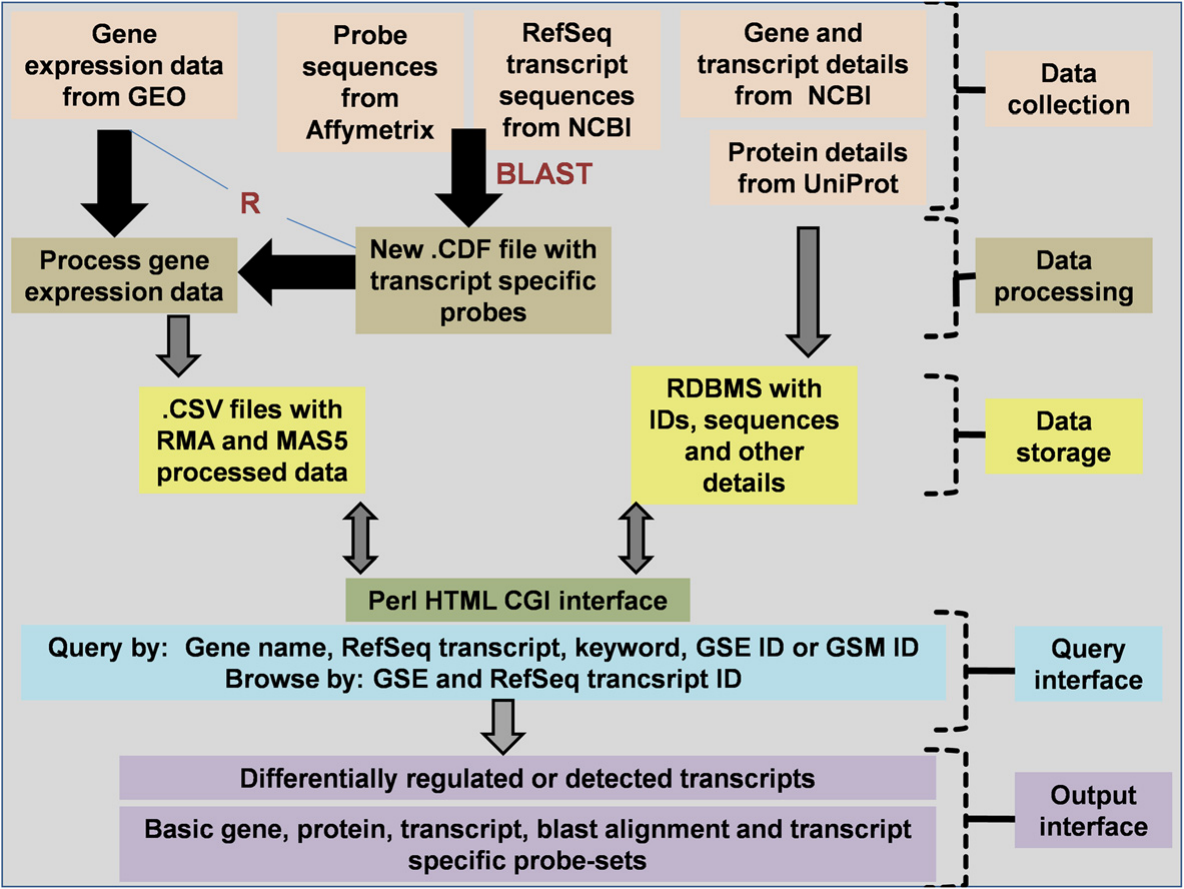
**Schematic representation of TIPMaP.** Description: The figure represents the steps involved in data collection, processing and storage as well as the details about the web interface.

An automated downloading and processing option is enabled for the selected, commonly used, 31 platforms. If the user selects GSEs that are not already downloaded, they will be downloaded and processed on priority and he/she will be intimated by an automated e-mail response. The tool’s schema is given in Figure 1.

## Results and discussion

### Data compiled

As of September 2013, data from 41,925 experiments were deposited in GEO. These include 31,034 gene expression profiling studies, of which 14,625 studies used Affymetrix chips. Affymetrix data from human (5954), mouse (4914) and rat (765) species formed the bulk. About 80 percentage of these experiments were performed using 31 (13 for human, 9 for mouse and 9 for rat) 3′ Affymetrix gene expression arrays. The other experiments include those done using 4 other 3′ Affymetrix gene expression arrays, which could not be included as the required CDF files were either modified or unavailable, and exon arrays - for which online tools [50] already exist. A list of all available platforms is available in the statistics section of the interface.

TIPMaP uses reanalyzed gene expression data, generated from the selected 31 platforms, to predict differentially regulated or detected transcripts. Currently the data correspond to 6000 biological conditions across 3 species. The tool provides expression data for 14670, 15621 and 12289 genes, and 17640, 15947 and 12355 RefSeq transcripts for human, mouse and rat species respectively (across all platforms, individual platforms details are available in the statistics section of the interface).

### Blast results

Only ‘good probes’ , i.e., those with 100% identity and coverage, were considered. Among such good probes, which formed 63% of the total probes, 68% matched uniquely to a specific transcript. Majority of them mapped to single-transcript-genes from NCBI RefSeq. For example, in the human genome U133 plus 2 array, 90% of transcript-specific probes corresponded to single-transcript-genes, while the remaining probes uniquely mapped to multi-transcript-genes [see Additional file 2, for distribution of transcript-specific probes across transcripts, genes & platforms]. These transcript specific probes were used to produce probe clusters specific to each transcript. Plenty of such probe-clusters indeed mapped uniquely to specific transcripts and majority of probe-clusters had three or more probes. For example, of the 14191 transcript specific probe-clusters from the human genome U133 plus 2 array, 492 had one probe each and 296 had 2 probes each, while 13403 had 3 or more probes in each cluster. These probe-clusters corresponded to 11933 single-transcript-genes and 2258 transcripts from multi-transcript-genes. Table 1 provides a glimpse of the probe-distribution across probe-clusters for 5 widely used platforms [see Additional file 3 for complete details across platforms]. Probe specificities to transcripts, for each platform, can be viewed in the statistics section of the interface. BLAST alignment results and transcript specific probe-clusters can be downloaded.

### Quality checks

Each step of the pipeline (aligning probes to transcripts, parsing blast result, identifying transcript specific probe-clusters, creating transcript specific CDF file, processing using R, retrieving and displaying of differentially regulated or detected alternatively spliced forms) was checked manually to ensure that the software works as intended.

Comparison of TIPMaP features with 14 other resources, reported earlier for similar applications, indicated that the newly developed tool is better than other resources [51].

### Experimental validation results

A set of 10 genes and 21 transcripts met the selection criteria (as described in the methods section). Expression pattern for these 21 transcripts were verified by RT-PCR [see Table 2]. Agreement pattern between TIPMaP and RT-PCR can be categorized in to three sections:

a) Complete agreement (57%): Eight transcripts found to be transcribed in all 31 hybridizations, with no ‘not-detected’ or ‘marginal’ calls, (indicated as 31-0-0) as per TIPMaP, were also transcribed in all 4 NOA samples as per RT-PCR. Similarly, 4 transcripts with a 100% profile of not being detected (0-31-0) in TIPMaP, were also not-detected in all samples.

b) Partial agreement/contradiction (29%): There were six transcripts which did not have a consistent detection call across 31 hybridizations (e.g., transcript NM_001160301.1 of DPYD gene had a profile of 1-30-0). Four of 6 such cases showed partial agreement. In the other two cases the experimental results contradicted the majority calls in TIPMaP.

c) Contradiction (14%): There was one transcript that was transcribed (31-0-0) in all 31 hybridizations as per TIPMaP, but was not-detected in all 4 NOA samples as per RT-PCR, which accounted to 4% of contradiction. There were two transcripts which were not-detected (0-31-0) in all 31 hybridizations as per TIPMaP, and were transcribed in all 4 NOA samples as per RT-PCR, which accounted to 10% contradiction. This 10% contradiction might be attributed to the higher sensitivity of RT-PCR compared to microarray.

**Table 2:** RT-PCR results for 10 genes and 21 transcripts

**Gene name**	RefSeq transcript ID (EPL)*	Gel images**	**RefSeq transcript ID (EPL)**	**Gel images**
DPYD	NM_000110.3 (161)		NM_001160301.1 (157)	
HNMT	NM_006895.2 (160)		NM_001024075.1 (142)	
PAFAH1B2	NM_001184748.1 (164)		NM_002572.3 (184)	
RPS6KA5	NM_182398.1 (84)		NM_004755.2 (165)	
EGFR	NM_005228.3 (179)		NM_201282.1 (143)	
			NM_201284.1 (151)	
TCF21	NM_003206.3 (186)		NM_198392.2 (212)	
KDELR3	NM_006855.2 (190)		NM_016657.1 (200)	
RALGPS1	NM_014636.2 (212)		NM_001190730.1 (162)	
GAD1	NM_000817.2 (204)		NM_013445.3 (149)	
GREB1	NM_014668.3 (166)		NM_033090.2 (172)	

*EPL: Expected product length, in nucleotides, for each transcript is mentioned in brackets.

**Gel images: lane 1: molecular weight marker, lanes 2-5: NOA samples 1-4 and lane 6: no template control.

The observed and expected product lengths were same in all cases.

Results from TIPMaP matched with RT-PCR in 70% of the cases (i.e., 58 out of 84 total experiments, 4 samples and 21 transcripts). When we considered only those transcripts with ‘transcribed’ or ‘not-detected’ status in all 31 hybridizations, the percentage agreement between the results was 80% [see Additional file 4 for RT-PCR and TIPMaP results comparative summary].

These results indicate that the new tool can be used to short-list transcripts, which are differentially regulated or detected in a tissue or a physiological condition, for further studies, using already existing microarray datasets. Molecular biologists and bioinformaticians might find various uses for the additional information provided by the tool. The additional information includes the consistency of expression type across hybridizations, the number of ‘good probes’ that uniquely map to the transcripts and position on the transcripts where these ‘good probes’ map.

## Conclusion

TIPMaP can be used to efficiently identify some of the differentially regulated transcripts from existing data generated using standard 3′ Affymetrix gene expression arrays. Even though further experimental validations are required for confirming the expression status of identified transcripts, the tool provides an easy way to make better use of available transcriptomics data across various mammalian tissues and conditions. The predictions by TIPMaP seem to be even more reliable in cases where the 100% hybridizations agree with the transcribed status, as indicated by 89% agreement during the current experimental validations. The tool aids in the differentiation of expression profiles with ‘higher reliability’ from those with ‘lower reliability’ based on their consistency of expression across hybridizations and number of transcript-specific probes used to predict the expression. Thus the tool can help to use the existing gene expression microarray data to short-list some of the transcripts with specific type of expression, across tissues and conditions, for further studies. It can also reduce the need for novel experiments involving human and animal samples from various tissues and conditions.

## Availability and requirements

**Project name:** TIPMaP

**Project home page:**http://resource.ibab.ac.in/TIPMaP

**Operating system(s):** Platform independent

**Programming language:** Perl based CGI and HTML scripts

**Other requirements:** Java

**Tested on:** Mozilla and Google chrome browsers

**Availability:** Free

## Abbreviations

GEO: Gene expression omnibus; NOA: Non-obstructive azoospermia; NCBI: National Center for Biotechnology Information; BLAST: Basic local alignment search tool; CDF: Chip definition file; RMA: Robust multi-array average; MAS5: Microarray Suite 5.0; PERL: Practical extraction and report language; HTML: Hypertext markup language; CGI: Common gateway interface; GSE: GEO series; GSM: GEO sample; GPL: GEO platform.

## Competing interests

The authors declare that they have no competing interests.

## Authors’ contributions

NC: Creation of transcript specific probe-clusters; gene expression data processing; design, creation and validation of the database; GB: Performed RT-PCR experiments; DSC: Gene expression data downloading & processing; also contributed to design and creation of the database; SA: Designed RT-PCR primers; VSS: Identified the donors with appropriate clinical conditions and collected the testicular NOA biopsy samples; KKA: Conceptualized the project, gathered the team, guided the work and supervised the progress. All authors read and approved the final manuscript.

## Authors’ information

Neelima Chitturi, Govindkumar Balagannavar, Darshan S Chandrashekar and Kshitish K Acharya: http://www.ibab.ac.in

Neelima Chitturi: http://www.iiit.ac.in

Darshan S Chandrashekar: http://www.manipal.edu/pages/welcome.aspx

Sadashivam Abinaya and Kshitish K Acharya: http://www.shodhaka.com

## Supplementary Material

Additional file 1 **Primers used for reverse transcription and polymerase chain reaction (RT-PCR), and the expected product lengths, for various transcripts.** The table includes gene name, transcript ID, forward and reverse primers used in RT-PCR experiments and expected product length. Click here for file

Additional file 2 **Distribution of transcript-specific probes across transcripts, genes and platforms.** The table describes the counts and percentages of transcript specific-probes for single-transcript-genes and multi-transcript-genes, for each platform. Click here for file

Additional file 3 **Distribution of transcript-specific probes and transcript-specific probe-clusters across platforms.** The table describes the distribution of transcript-specific probes and of transcript-specific probe-clusters for single-transcript-genes and multi-transcript genes, across platforms. Click here for file

Additional file 4 **Summary of RT-PCR and TIPMaP comparisons.** The details include gene name, detection status from MGEx-TDB, detection status and hybridization summary from TIPMaP and agreement between TIPMaP and RT-PCR results. Click here for file
